# Aryl hydrocarbon receptor nuclear translocator (ARNT) gene as a positional and functional candidate for type 2 diabetes and prediabetic intermediate traits: Mutation detection, case-control studies, and gene expression analysis

**DOI:** 10.1186/1471-2350-9-16

**Published:** 2008-03-17

**Authors:** Swapan K Das, Neeraj K Sharma, Winston S Chu, Hua Wang, Steven C Elbein

**Affiliations:** 1 Division of Endocrinology and Metabolism, Department of Medicine, College of Medicine, University of Arkansas for Medical Sciences, Little Rock, Arkansas, USA; 2 Endocrinology Section, Medicine and Research Services, Central Arkansas Veterans Healthcare System, Little Rock, Arkansas, USA

## Abstract

**Background:**

ARNT, a member of the basic helix-loop-helix family of transcription factors, is located on human chromosome 1q21–q24, a region which showed well replicated linkage to type 2 diabetes. We hypothesized that common polymorphisms in the *ARNT *gene might increase the susceptibility to type 2 diabetes through impaired glucose-stimulated insulin secretion.

**Methods:**

We selected 9 single nucleotide polymorphisms to tag common variation across the *ARNT *gene. Additionally we searched for novel variants in functional coding domains in European American and African American samples. Case-control studies were performed in 191 European American individuals with type 2 diabetes and 187 nondiabetic European American control individuals, and in 372 African American individuals with type 2 diabetes and 194 African American control individuals. Metabolic effects of *ARNT *variants were examined in 122 members of 26 European American families from Utah and in 225 unrelated individuals from Arkansas. Gene expression was tested in 8 sibling pairs discordant for type 2 diabetes.

**Results:**

No nonsynonymous variants or novel polymorphisms were identified. No SNP was associated with type 2 diabetes in either African Americans or European Americans, but among nondiabetic European American individuals, *ARNT *SNPs rs188970 and rs11204735 were associated with acute insulin response (AIR_g_; p =< 0.005). SNP rs2134688 interacted with body mass index to alter β-cell compensation to insulin resistance (disposition index; p = 0.004). No significant difference in *ARNT *mRNA levels was observed in transformed lymphocytes from sibling pairs discordant for type 2 diabetes.

**Conclusion:**

Common *ARNT *variants are unlikely to explain the linkage signal on chromosome 1q, but may alter insulin secretion in nondiabetic subjects. Our studies cannot exclude a role for rare variants or variants of small (< 1.6) effect size.

## Background

Type 2 diabetes mellitus is a complex heterogeneous group of metabolic conditions characterized by elevated levels of serum glucose. Impairment in both hepatic and peripheral insulin action, and defective pancreatic insulin secretion characterize fully developed type 2 diabetes. The interaction between genetic risk factors and environmental or lifestyle factors likely account for the current rising prevalence of type 2 diabetes among many populations, including United States European Americans and African Americans [1].

Aryl Hydrocarbon Receptor Nuclear Translocator (ARNT, also known as hypoxia inducible factor-1β) is a member of the basic helix-loop-helix Per/AhR/ARNT/Sim (bHLH-PAS) family of transcription factors. ARNT and its interacting partners HIF1α, HIF2α, and AhR form heterodimeric complexes which are required for cellular responses to hypoxia and environmental toxins such as dioxin. ARNT complexes also directly regulate the expression of genes involved in glucose transport and glucose metabolism [2]. Recently, Gunton et al [3] implicated ARNT directly in impaired insulin secretion in type 2 diabetes by showing a 90% decrease *ARNT *messenger RNA in islets from diabetic individuals when compared to normal islets. Down-regulation of *ARNT *expression decreased the glucose stimulated insulin secretion in the Min 6 β-cell line, and mice lacking β-cell *ARNT *expression exhibited abnormal glucose tolerance, impaired glucose stimulated insulin secretion, and changes in islet gene expression similar to human type 2 diabetes [3]. These studies have implicated ARNT in the transcriptional regulation of genes required for optimal glucose-responsive insulin secretion, and thus suggest *ARNT *as a strong candidate gene for diabetes. A further role of ARNT in type 2 diabetes is suggested by its role as an obligate partner of several transcription factors involved in the response to toxins and hypoxic stress [4]. Hence, ARNT may integrate the genetic predisposition to β-cell failure [5] with environmental insults leading to diabetes pathogenesis.

The *ARNT *gene is located on human chromosome 1q21.2, a region with well replicated linkage to type 2 diabetes by us in European American and African American populations, and by others in European American, Chinese, and Pima Indian populations [6]. Thus, in addition to being a strong functional candidate, *ARNT *is a strong positional candidate gene for type 2 diabetes. We hypothesized that common polymorphisms in the *ARNT *gene would alter its function or expression, and in turn increase the susceptibility to diabetes by affecting glucose-stimulated insulin secretion. We evaluated the association of common *ARNT *sequence variants with type 2 diabetes by using HapMap phase2 linkage disequilibrium information [7] as well as by screening putative functional regions of the gene for sequence variants in European American and African American subjects, and by testing these variants for an association with type 2 diabetes. To examine the physiologic impact of *ARNT *variants, we studied 352 non-diabetic subjects who had undergone frequently sampled intravenous glucose tolerance tests, including members of families ascertained in Utah and unrelated European American subjects ascertained in Arkansas. We also examined *ARNT *expression in transformed lymphocytes as a surrogate tissue using a discordant sib pair design, and we searched for additional evidence for *cis*-acting regulatory sequence polymorphisms that would alter the mRNA expression ratio between *ARNT *alleles.

## Methods

### Study subjects

Phenotypic features of our study populations are summarized in the Table 1. Case-control studies with type 2 diabetes were conducted primarily in two samples: a European American cohort of 191 diabetic subjects and 187 control subjects, and in an African-American cohort of 372 type 2 diabetic patients and 194 control individuals. European American individuals were ascertained in Utah for Northern European ancestry, as described previously [8,9], with additional control individuals ascertained in Arkansas for similar criteria. All African American individuals were ascertained in Arkansas. For all case-control cohorts, individuals with diabetes had known type 2 diabetes on pharmacological therapy, or had a documented high fasting glucose or diabetic glucose tolerance test. All diabetic individuals and had at least one other first degree relative with type 2 diabetes. Non-diabetic control individuals had no history of diabetes in a first degree relative and had either a normal 75 g oral glucose tolerance test or a normal fasting glucose level (below 5.6 mM). Replication of case-control association was performed in a European American cohort of 240 T2DM and 170 control subjects ascertained in Arkansas for similar selection criteria. Because of some overlap in controls with the initial case control study, the combined study size was 315 controls and 429 cases. All of our case individuals were recruited from diabetes clinics. African American diabetic individuals were recruited in large part from Department of Veterans Affairs diabetes clinics, resulting in a predominance of male diabetic individuals. Control individuals were recruited from spouses or family members of cases, by public advertisement, and by flyers posted in hospitals, clinics, and other public locations. Many control subjects were participants in other ongoing studies that recruited nondiabetic subjects without a family history of diabetes. Because female volunteers are in excess in most studies, our control population was skewed toward women whereas our case population was skewed toward men.

**Table 1:** Summary of Study Populations

**Population**	**Description**	**Male/Female**	**BMI(Kg/m2)**	**Age(years)**	**Diagnosis age**
Utah Caucasian case/control cohort1	Control	72/115	27.5 (18.5,41.1)	51.0 ± 15.3	---
	Case	134/57	31.1 (21.9,44.2)	61.7 ± 10.7	51.5 ± 12.1
AR Caucasian case/control cohort2	Control	65/105	28.1 (19.1, 41.2)	41.0 ± 14.4	---
	Case	166/74	31.78 (21.7, 46.5)	60.5 ± 12.2	49.9 ± 12.8
African American case/control cohort	Control	96/98	29.5 (18.8,46.3)	42.8 ± 13.3	---
	Case	198/174	32.0 (20.8, 49.4)	54.8 ± 12.5	42.8 ± 11.9
Utah Caucasian metabolic	Family based non-diabetic	50/72	27.5 (18.3,41.3)	39.3 ± 10.5	---
AR Caucasian metabolic	Population based non-diabetic	81/149	29.4 (19.8, 43.7)	37.2 ± 9.5	---

Metabolic effects of *ARNT *sequence polymorphisms were studied in two nondiabetic populations: a family based cohort of 122 non-diabetic members from 26 families of Northern European descent ascertained in Utah [10,11], and a European American population of 225 unrelated nondiabetic subjects ascertained in Arkansas with age under 60 years. All Utah family members underwent a tolbutamide – modified, frequently sampled intravenous glucose tolerance test (FSIGT). Because tolbutamide became unavailable during the study, 100 subjects had tolbutamide modified tests, whereas the remainder had an insulin modified (0.04 unit/kg) FSIGT [11]. All subjects provided written, informed consent under a protocol approved by the Institutional Review Board of either the University of Utah Health Sciences Center or the University of Arkansas for Medical Sciences.

### Genetic analysis

**Figure 1:**
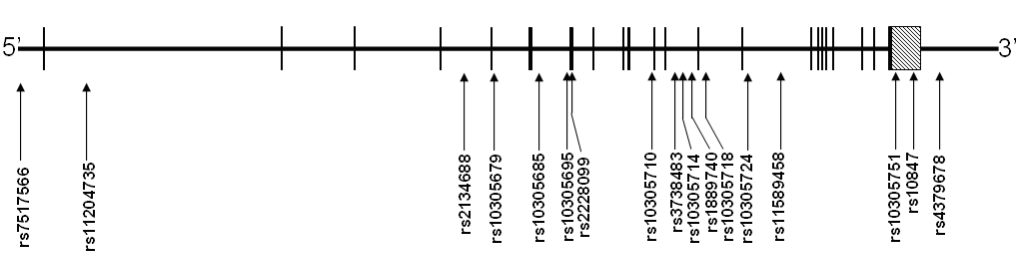
**Structure of the *ARNT *gene and location of SNPs analyzed**. The approximate location of all 22 reference sequence exons is shown as black bars, and the large variable 3'UTR region is shown as hatched box. The location of the SNPs is shown as arrows below the schematic. Linkage disequilibrium relationships are shown in Figure 2 and 3.

**Figure 2:**
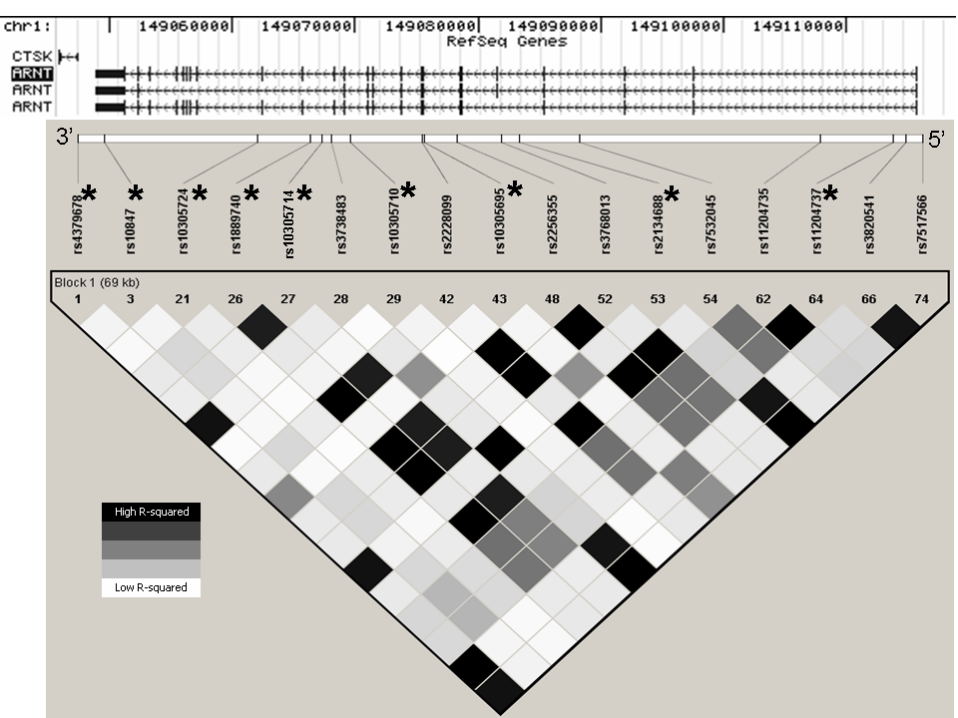
**Linkage disequilibrium pattern of the ARNT region based on HapMap data**. Markers selected for genotyping in our case-control cohorts are marked by an asterisk (*). The top portion of each diagram shows the known exon-intron structure. Only SNPs with a minor allele frequency over 5% in HapMap data were selected to construct the linkage disequilibrium map. Figure 2 shows linkage disequilibrium based on the r^2 ^statistic using Hapmap Caucasian (CEPH) data.

**Figure 3:**
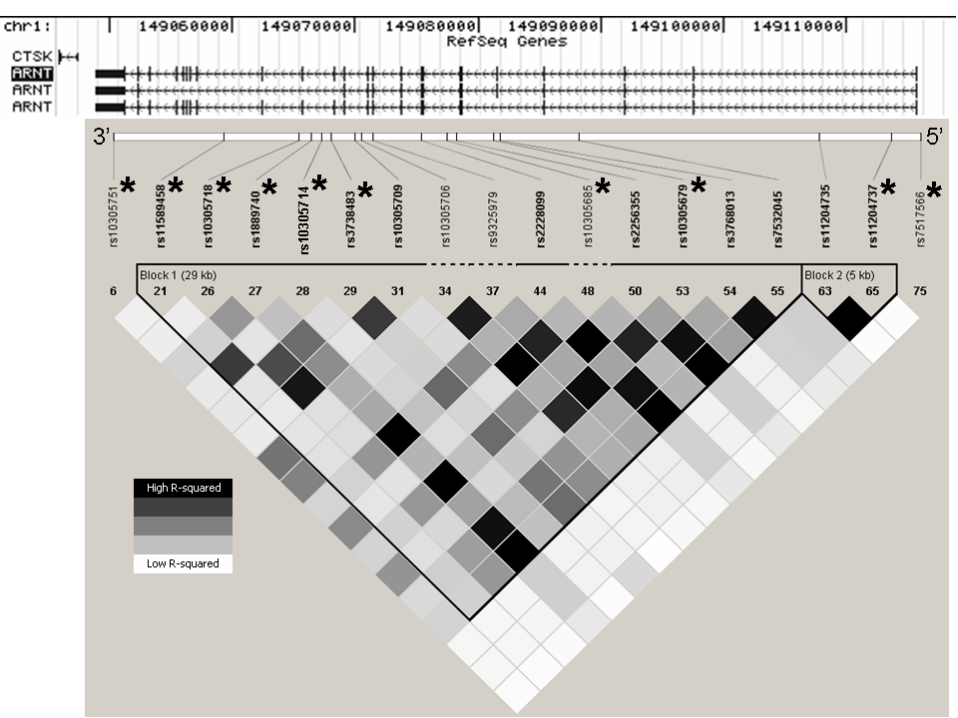
**Linkage disequilibrium pattern of the ARNT region based on HapMap data in Yorubans**. Figure 3 is identical to Figure 2, except shown in Yorubans for selection of SNPs in African Americans. Asterisks (*) identify markers selected for genotyping.

The *ARNT *gene spans ~67 kb of human chromosome1q21.2 and consists of 22 exons (Figure 1). We examined 74 single nucleotide polymorphisms (SNPs) from the August 2006 HapMap data [7] for the *ARNT *gene and 2 kb of surrounding sequence. We identified 17 SNPs that were both polymorphic and had minor allele frequencies over 5%, from which we selected 9 tagSNPs using the TAGGER program as implemented in HaploView 3.2 [12] with parameters of r^2 ^≤ 0.9 (see Figure 2). Similarly, from 75 HapMap SNPs reported in the Yoruban population of West Africa (Nigerian), we identified 18 SNPs that were both polymorphic and had a minor allele frequency exceeding 5% (Figure 3). We forced the inclusion of the 3 SNPs that were also polymorphic in European Americans, and identified an additional 7 tagSNPs (r^2 ^≤ 0.9, TAGGER program) to cover common variation in the *ARNT *gene in an African-American population (Figures 2 and 3). SNPs were genotyped by Pyrosequencing (PSQ96, Biotage AB, Uppsula, Sweden) using a modification of manufacturer's protocol that used a biotinylated universal primer [9]. SNPs showing an association with type 2 diabetes in the initial Northern European case-control cohort were genotyped in a confirmatory Arkansas European American case-control cohort. Associated SNPs and any SNPs with a minor allele frequency over 10% in European Americans were also tested for an association with quantitative prediabetic traits in non-diabetic individuals.

### Screening for novel variants

Given the large size of the *ARNT *gene, we focused the screening for novel variants on the known major functional domains: the DNA binding basic helix loop helix (bHLH) domain (amino acid residues 103–143); the dimerization (PAS) domain (amino acid residues 161–235 and 349–419); and the transactivation domain at the carboxyl terminal. Primers were designed for amplicons of under 600 bp using sequence NM_001668 and the University of California Santa Cruz (genome.ucsc.edu) genome and proteome browsers for exons 1 (amino terminal), 6 (bHLH domain), 7 (PAS domain 1), 12–13 (PAS domain 2), and 22 (transactivation domain). Additionally, the flanking intronic sequences, 5' untranslated region, and 272 bp of the 3' untranslated region were screened. Fragments were screened using denaturing high-performance liquid chromatography (DHPLC) on a Transgenomic WAVE HT DNA fragment analysis system (Transgenomic Inc., Omaha, NE) in 24 European American and 24 African American individuals, each population comprising 16 diabetic and 8 nondiabetic participants. Variant chromatographic patterns were characterized by dideoxy DNA sequencing. The 2 kb region 5' to the transcription start site, which was non amenable to DHPLC screening, was sequenced directly in the same 48 individuals (Polymorphic DNA Technologies, Inc.; Alameda, CA).

### Gene expression

Transformed lymphocytes from 8 sibling pairs discordant for type 2 diabetes were grown under normoglycemic (5.6 mM glucose) conditions, then exposed to either 5.6 mM glucose, or 28 mM glucose and 5 nM insulin for 8 hours before harvest. Total RNA was extracted using RNEasy mini kit (Qiagene Inc., Valancia, CA), the quantity and quality checked using an Agilent 2100 Bioanalyzer, and 600 ng reverse transcribed using random hexamer primers (TaqMan Reverse Transcription Reagents, Applied Biosystems, Inc). *ARNT *expression was measured by real time PCR on a Rotorgene 2000 Real time-PCR system (Corbett Life Science, Sydney Australia), with 18S ribosomal RNA as a normalization standard. Primer sequences were as follows: 18S forward: ATCAACTTTCGATGG TAGTCG, 18S reverse: TCCTTGGATGTGGTAGCCG, ARNT – Forward: TTCATCCCATACTCAAAATACCC, ARNT – Reverse: AAAGCAAAACCCAATCTCAA.

### Allelic expression imbalance

Unequal expression of *ARNT *alleles was sought as evidence for *cis *acting regulatory variants by comparing peak heights in individuals heterozygous for the synonymous coding SNP rs2228099 or 3' untranslated SNP rs10847, using methods described elsewhere [13]. Briefly, total RNA was reverse transcribed using random hexamers. Allelic specific quantitation of both cDNA and genomic DNA samples was determined using the same assay for pyrosequencing on a PSQ 96 Pyrosequencer (Biotage, Inc, Uppsala, Sweden), with peak height quantified using Allele Quantification software (Biotage, Inc).

### Statistical analysis

Allele frequencies in case and control populations were compared using Fisher Exact and Cochran-Armitage Trend tests. Hardy-Weinberg equilibrium was tested using the online DeFinetti program [14]. Linkage Disequilibrium and haplotype association analyses were performed by Haploview v. 3.32 [12]. Insulin secretion was calculated as the acute insulin response to glucose (AIR_g_), determined either as the mean excursion over baseline from 2–10 min (Utah population) or the 2 min – 10 min Area Under Curve (AUC; Arkansas population). Although they give different values, the two methods of computing AIR_g_are highly correlated. Insulin sensitivity (S_I_) was calculated from the FSIGT using either the MinMod (Utah sample) or MinMod Millenium (Arkansas Sample) programs [15,16]. The two programs use the same algorithms and provide nearly identical estimates of S_I_. The ability of the β-cell to compensate for insulin sensitivity was determined by the disposition index (DI = S_I _*AIR_g_) [17,18]. Genotypic effects on glucose homeostasis traits (S_I_, AIR_g_, DI) were tested using mixed effect, general linear regression models implemented in SPSS v.12 for Windows (SPSS Inc., Chicago, IL). Skewed variables were ln-transformed to normality prior to analysis, and age, body mass index (BMI), gender, genotype, protocol (tolbutamide or insulin), and diagnosis (IGT or glucose tolerant) were included as factors and covariates, as appropriate. Pedigree membership was included as a random factor in analyses of family-based Utah samples.

*ARNT *expression was normalized to 18S RNA and both pair-wise and group-wise differences compared using the nonparametric Mann-Whitney U and Wilcoxan signed rank tests. For allelic expression imbalance, we computed the 95% CI for the assay from DNA (expected ratio 1:1). We considered allelic expression imbalance to be present if the *ARNT *RNA ratio fell outside the 95% CI for DNA in heterozygous individuals. The significance for the number of samples falling outside the 95% CI was determined using a chi-square goodness of fit test.

### Power considerations

We calculated the power assuming p < 0.05 and for odds ratios over 1.5 (approximately that of TCF7L2) using allelic association (approximately the same as the Cochran Armitage trend test). We considered a control minor allele frequency range of 0.1 to 0.4. Among our primary European American population, we had 70% power at an odds ratio of 1.7 for a minor allele frequency of 0.1, or over 70% power for an odds ratio of 1.6 or greater for a minor allele frequency of 0.15 – 0.25, and over 70% for a minor allele frequency over 0.25 at an odds ratio of 1.5. Among African Americans, our power exceeded 70% for an odds ratio over 1.5 at minor allele frequencies over 0.15, but would have required an odds ratio of 1.6 for 70% power at a minor allele frequency of 0.1.

## Results

### Association with type 2 diabetes

Results of both European American and African American association studies are shown in Table 2, with raw genotype data shown in Table 3. No individual SNP was associated with type 2 diabetes in either population, with the best trend for rs10305685 in African Americans (p = 0.067; OR = 0.75). In the European American case-control study, SNPs rs2134688 (intron 4) and rs4379678 (3'flanking) were in complete linkage disequilibrium (r^2 ^= 1) despite selection on r^2 ^< 0.9. Excess homozygosity for the minor allele was observed in controls for both SNPs, with evidence for a protective effect of the rare homozygous alleles (p = 0.006; Table 3). We expanded SNP rs2134688 into an independent European American case-control cohort ascertained in Arkansas, where no trend to an association with type 2 diabetes was observed (Table 3).

**Table 2:** Summary of single nucleotide polymorphisms and allele frequencies

**Position**	**RS Number**	**Genome Location**	**Variant**	**Gene Location**	**Caucasian Case/Control Frequency**	**African – American Case/Control Frequency**
-992	rs7517566	149116659	C/T	5' Flanking	ND^1^	0.138/0.17
7377	rs11204735	149108291	G/A	Intron 1	0.432/0.448	0.164/0.169^3^
32158	rs2134688	149083510	T/C	Intron 4	0.087/0.086	NP
34242	rs10305679	149081426	T/C	Intron 5	NP	0.194/0.221
38039	rs10305685	149077629	A/G	Intron 6	NP	0.158/0.201
39928	rs10305695	149075740	G/A	Intron 6	0.032/0.041	NP
40155	rs2228099	149075513	G/C	Exon 7 (Val to Val)	ND^2^	ND^2^
46017	rs10305710	149069651	G/A	Intron 10	0.041/0.059	NP
47578	rs3738483	149068090	G/A	Intron 12	ND^1^	0.322/0.348
48389	rs10305714	149067279	T/C	Intron 12	0.324/0.344	0.265/0.232
49289	rs1889740	149066379	G/A	Intron 12	0.345/0.363	0.481/0.466
50312	rs10305718	149065356	C/G	Intron 13	NP	0.189/0.173
53679	rs10305724	149061989	C/T	Intron 14	0.061/0.041	NP
56428	rs11589458	149059240	T/C	Intron 14	ND	0.22/0.192
65586	rs10305751	149050082	G/A	3'UTR	< 0.05	0.124/0.133
66247	rs10847	149049421	G/A	3'UTR	0.28/0.319	< 0.05
68462	rs4379678	149047206	A/G	3' Flanking/Intron 1 of CTSK	0.087/0.085	NP

Position is given relative to the A of the ATG start site. Genome location is based on the March 2006 (hg18) build. Variant is given for the negative (coding) strand as major/minor allele in Caucasian samples. Frequencies are given for the minor allele in Caucasian and African American samples. NP, not polymorphic in HapMap dataset; ND, not done. ^1 ^r^2 ^= 1 with rs2134688; ^2^, r^2 ^= 1 with rs1889740; ^3 ^minor allele is "G" in African Americans.

**Table 3:** Raw counts of cases and controls by genotype for *ARNT *region SNPs in Caucasians and African Americans

**Position**	**RS number**	**Population**	**Variant**	**Control**			**Type 2 Diabetes**		
				
				**DD**	**Dd**	**dd**	**DD**	**Dd**	**dd**
-992	rs7517566	AA	C/T	132	53	6	276	86	8
7377	rs11204735	UT AA	G/A A/G	60 129	81 61	41 2	60 260	96 102	34 10
32158	rs2134688	UT AR	T/C T/C	158 167	17 24	7 2	157 176	33 37	0 3
34242	rs10305679	AA	T/C	116	64	10	243	112	16
38039	rs10305685	AA	A/G	122	63	7	264	95	11
39928	rs10305695	UT	G/A	169	11	2	179	12	0
46017	rs10305710	UT	C/T	158	19	1	171	11	2
47578	rs3738483	AA	C/T	84	81	26	175	152	43
48389	rs10305714	UT AA	T/C T/C	82 114	74 65	26 12	86 201	82 142	20 27
49289	rs1889740	UT AA	C/T C/T	78 58	76 87	28 45	83 102	83 179	24 88
50312	rs10305718	AA	G/C	130	56	5	247	108	16
53679	rs10305724	UT	C/T	170	9	3	168	23	0
56428	rs11589458	AA	T/C	128	56	9	230	119	22
65586	rs10305751	AA	C/T	142	49	1	289	74	9
66247	rs10847	UT	C/T	88	74	21	102	68	19
68462	rs4379678	UT	T/C	158	17	7	157	33	0

Position is shown relative to ATG start site. Variants are shown for the major/minor allele, as genotyped. Populations are as described in Table 1: UT, Utah Caucasian; AR, Arkansas Caucasian; AA, African-American. The total number of samples genoytped varies due to genotyping success rates, and may differ from the totals in Table 1.

Screening of the *ARNT *gene for additional variants identified a single synonymous SNP in exon 7, previously reported as rs2228099. Our selected tagSNP rs1889740, which showed no association with type 2 diabetes in European American or African American populations, was a perfect proxy (r^2 ^= 1) in both HapMap CEPH and Yoruban populations. We confirmed the strong linkage disequilibrium in 120 European American individuals. We confirmed also the presence of common SNPs rs7517566 in the 5' flanking region, rs35756904 in the 5' untranslated region, rs10305650 in intron 1, rs2256355 and rs1027699 in intron 6, rs3738483 and rs3768017 in intron 12,, and rs10305749 in the 3' untranslated region. Each common variant was typed directly or tagged by a typed SNP at r^2 ^> 0.9, and neither the typed SNPs nor excellent proxies were associated with type 2 diabetes. We also identified a very rare SNP (rs7515228; 1 heterozygous individual in 48 samples) in the 5' flanking region, which was not typed due to low power in our population.

In European Americans, 8/9 SNPs were confirmed to have a minor allele frequency over 5%, and all 8 SNPs fell within a single block using the solid spline block definitions [12]. The 8 SNPs defined 7 common haplotypes, of which none differed significantly in frequency between cases and controls (Table 4). Similarly, among the African American population all 10 tagSNPs fell within a single block. Haplotype "CTGTTCGCAC" was more common among controls (6.7%) than among cases (3.6%), as was a 4 marker sliding window (ATAC; 50.3% in cases vs 43.8% in controls, p = 0.038). Neither nominal association remained significant on permutation testing (Table 5).

**Table 4:** Haplotype Frequencies for ARNT SNPs in Utah Caucasian Cases and Controls

**Haplotype**	**Case Frequency**	**Control frequency**	**P Value**
TCCTCCTA	0.324	0.340	0.624
TTCCTCTG	0.282	0.319	0.280
TCCCTCTG	0.185	0.133	0.055
CCCCTCCA	0.087	0.085	0.935
TCTCTCTG	0.061	0.041	0.232
TCCCTTTG	0.041	0.056	0.324
TCCTTCTA	0.021	0.022	0.928

The 8 marker haplotypes are shown for all markers with minor allele frequency over 5%. The bases are shown according to the assay and may be the complement to the nucleotide in the public database. The SNPs included were rs4379678, rs10847, rs10305724, rs1889740, rs10305714, rs10305710, rs2134688, rs11204735.

**Table 5:** Haplotype frequencies for ARNT SNPs in African American cases and controls

**Haplotype**	**Case Frequency**	**Control Frequency**	**P Value**
ATAC	0.503	0.438	**0.038**
GCAC	0.157	0.199	0.076
ATGC	0.164	0.170	0.820
ATAT	0.138	0.170	0.163
ACAC	0.037	0.023	0.224

Four marker haplotypes are shown using markers rs10305685, rs10305679, rs11204735, and rs7517566. Haplotypes are shown with the nucleotide as genotyped in the assay, which may differ from the nucleotide listed in public databases.

### *ARNT *SNPs and insulin secretion

Based on the proposed role of *ARNT *in insulin secretion [3], we selected 4 SNPs that showed a trend to an association with type 2 diabetes or that had a minor allele frequency over 10% to test in two populations of nondiabetic European American individuals who had undergone detailed phenotyping of insulin sensitivity and secretion. Both rs188970 (p = 0.005) and rs11204735 (p = 0.002) were associated with AIR_g _in nondiabetic members of high risk Utah families. However, neither finding was replicated in and unrelated European American sample from Arkansas. Disposition index (DI), a measure of the ability of the β-cell to compensate for insulin resistance, was associated with rs2134688 in unrelated individuals from Arkansas in interaction with both body mass index (p = 0.004) and age (p = 0.04). Marginal means for all analyses are shown in Table 6. Because *ARNT *null mice showed sexual dimorphism in effects on insulin secretion, we also tested for an interaction of genotype with gender. Rs2134688 showed a significantly lower mean DI among women (0.228) than men (4.822; p = 0.001) among Utah family members, but this interaction was not observed in individuals from Arkansas (data not shown).

**Table 6:** Marginal means for intravenous glucose tolerance measures

**SNP**		**Utah Caucasian Family samples**				**Arkansas Caucasian population**			
**rs10847**		**CC**	**CT**	**TT**	**p-value**	**CC**	**CT**	**TT**	**p-value**
				
	**n**	68	47	10		124	85	21	
	
	**S_I_**	5.66 (4.29,7.48)	6.14 (4.64,8.11)	7.75 (1.84,32.64)	0.84	4.85 (4.11, 5.71)	5.45 (4.47, 6.63)	5.42 (3.58, 8.18)	0.64
	**AIRg**	116 (93,145)	152 (122,190)	249 (81,757)	0.09	2370 (2082,2694))	2142 (1830,2502)	1890 (1380,2580)	0.32
	**DI**	0.99 (0.59,1.66)	1.29 (0.77,2.16)	1.55 (0.42,5.68)	0.5	1152 (969,1371)	1173 (951,1447)	1111 (716,1725)	0.37

**rs188970**		**CC**	**CT**	**TT**	**p-value**	**CC**	**CT**	**TT**	**p-value**
				
	**n**	48	51	23		87	103	32	
	
	**S_I_**	5.29 (4.11,6.81)	6.40 (4.83,8.46)	6.45 (4.27,9.73)	0.49	5.68 (5.07, 6.37)	5.40 (4.85, 6.00)	5.70 (4.68, 6.93)	0.11
	**AIRg**	**200 (163,246)**	**141 (113,177)**	**115 (82,159)**	**0.005**	2388 (2046,2784)	2082 (1806,2400)	2664 (2052, 3468)	0.18
	**DI**	1.25 (0.76,2.06)	1.13 (0.65,1.97)	1.07 (0.47,2.42)	0.91	1356 (1170,1573)	1122 (979,1287)	1520 (1180,1958)	0.37

**rs2134688**		**TT**	**TC+CC**		**p-value**	**TT**	**TC+CC**		**p-value**
				
	**n**	96	26			193	36		
	
	**S_I_**	6.62 (6.21,8.41)	5.80 (3.56,9.45)		0.86	5.48 (5.05,6.37)	5.38 (4.43, 6.52)		0.86
	**AIR_g_**	164 (134, 200)	132 (88, 197)		0.31	2244 (2022,2490)	2280 (1788,2910)		0.66
	**DI**	1.33 (0.91,1.95)	1.07 (0.59,1.93)		0.5	1132 (987,1299)	1201 (867,1633)		0.94

**rs11204735**		**GG**	**GA**	**AA**	**p-value**	**GG**	**GA**	**AA**	**p-value**
				
	**n**	34	53	31		66	115	48	
	
	**S_I_**	5.49 (3.90,7.72)	6.28 (4.70,8.39)	4.92 (3.61,6.71)	0.46	5.60 (4.88, 6.45)	5.22 (4.70, 5.78)	5.52 (4.68, 6.52)	0.66
	**AIRg**	**117 (89,152)**	**150 (119,188)**	**216 (169,277)**	**0.002**	2370 (1986,2832)	2220 (1944,2538)	2190 (1776,2700)	0.8
	**DI**	0.71 (0.38,132)	1.36 (0.81,230)	1.36 (0.78,2.38)	0.14	1328 (1112,1587)	1157 (1012,1322)	1209 (980,1492)	0.46

Marginal means are shown after adjustment for age, gender and BMI; significance is based on a general linear model and p values are shown without correction for multiple testing. AIR_g _in the Utah Caucasian study is from the mean 2–10 min post challenge insulin excursion in pmol/l; for Arkansas Caucasian study, the AIR_g _is taken from MinMod Millenium output and is the area under the curve from 0 to 10 min, converted to pmol/l. All means are transformed back to the linear scale from Ln-transformed values; 95% confidence intervals are provided in parenthesis. S_I_, insulin sensitivity index from MinMod in (pmol/l) min^-1^; AIR_g_, acute 2–10 min insulin response to intravenous glucose bolus, in pmol/l (UT sample) or pmol-min/l (AR sample); DI, Disposition index (S_I _× AIR_g_) has no units. Values from the Utah sample are × 10^-2^. Note that the MinMod Millenium program calculates disposition index using the area under curve for AIR_g _and without the decimal places for S_I _(10^-5 ^(pmol/l) min^-1^. Because calculations in the Utah population used mean 2 min – 10 min insulin to calculate AIR_g _and calculated S_I _× AIR_g _values were calculated using the legacy MinMod program and Arkansas samples using the MinMod Millenium program, the values between populations are not directly comparable.

### *ARNT *gene expression

*ARNT *mRNA levels in transformed lymphocytes did not differ between 8 European American control individuals and cell lines from their 8 type 2 diabetic siblings, either when grown under normoglycemic conditions (*ARNT *to 18 S RNA ratio 0.958, range 0.611–1.285 in controls *vs *1.067, range 0.446 – 1.555 in diabetic cell lines; p = 0.40) or when cultured in 28 mM glucose and 5 nmol/l insulin (0.8131.067, range 0.449–1.164 in controls; 0.946, range 0.400 – 2.329 in type 2 diabetes; p = 0.67).

**Figure 4:**
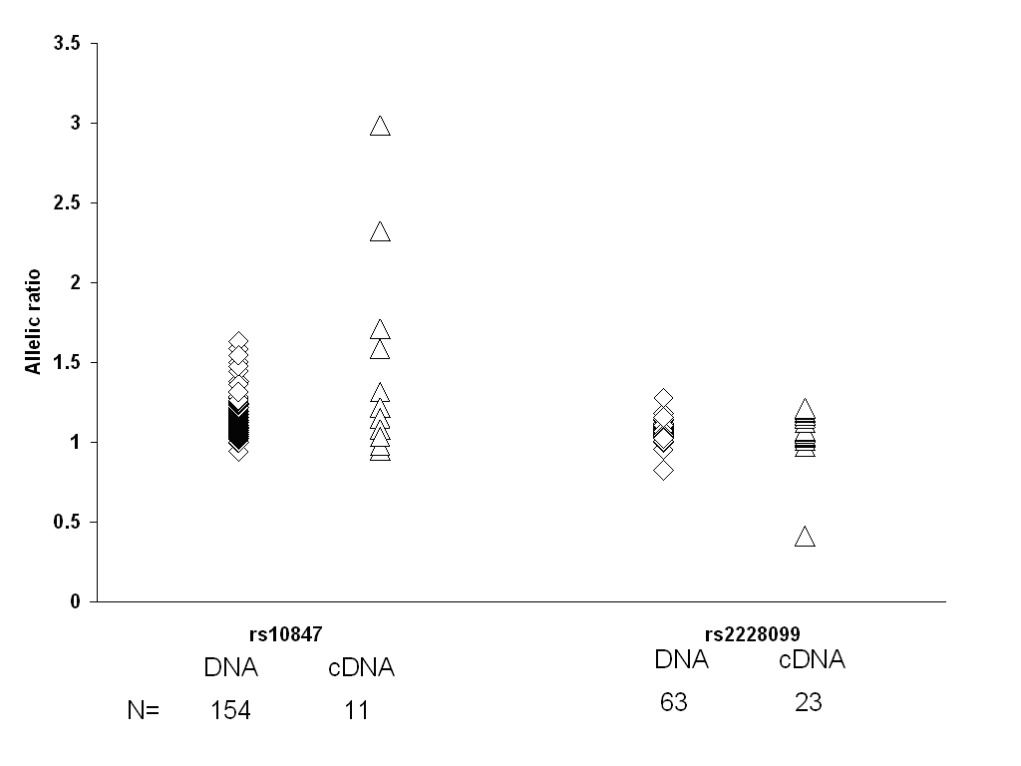
**Allelic expression imbalance at 3'UTR (rs10847) and exonic (rs2228099) SNPs of the *ARNT *gene**. The scatter plot shows the allele specific expression in cDNA (△) as compared to genomic DNA (◇) for 11 heterozygous samples at rs10847 (C/T) and 23 heterozygous samples at rs2228099 (C/G).

We searched for evidence of *cis*-acting regulatory variants by testing the ratio of *ARNT *transcripts (cDNA) in transformed lymphocytes from European American and African American individuals who were heterozygous for the transcribed variants rs2228099 and rs10847 (Figure 4). We compared cDNA from 11 individuals at rs10847 to 154 DNA samples, and 23 cDNA samples at rs228099 to 63 DNA samples. Both SNPs showed some evidence of allelic expression imbalance, with 4/11 samples for rs10847 and 7/23 for rs228099 falling outside of the 95% confidence intervals for the allelic ratio set from the cDNA (p = 0.0001 and p = 0.001, respectively). At rs10847, samples showed allelic ratios of 1.6 to 3.0 (expected 1.14 from DNA), whereas deviations from expectations were modest for rs228099 (ratios 1.18–1.21, expected 1.07; Figure 4). A single sample at rs228099 showed a ratio in the opposite direction (ratio 0.41).

## Discussion

ARNT (or HIF1β) is the obligate heterodimer for a family of bHLH-PAS transcription factors that include HIF1α, HIF2α and AhR. These proteins in turn mediate signal transduction in response to both hypoxia and environmental toxins, including dioxin and polycyclic aromatic hydrocarbons [3]. Recent data have also implicated ARNT in regulation of important downstream genes in the β-cell, including HNF4α, IRS2, AKT2, and enzymes of the glycolytic pathway [3]. Indeed, putative ARNT binding sites may exceed 13,000 in the genome [3], and may include genes involved in glucose metabolism, vascular function, and oxygen transport (erythropoietin). Genes downstream of ARNT such as *HNF4α *may in turn bind to large numbers of promoters [19]. In addition to data supporting *ARNT *as a strong functional candidate gene for type 2 diabetes, the *ARNT *gene is located on human chromosome 1q21, a region with replicated linkage to type 2 diabetes in diverse populations. These facts suggested that genetic variants altering *ARNT *function or regulation would impact glucose homeostasis and diabetes risk. To test this hypothesis we searched for indirect evidence that SNPs marking common haplotypes were associated with type 2 diabetes or altered insulin secretion. We also searched directly for coding or highly conserved variants that might directly impact ARNT function.

In the current study, we found no nonsynonymous SNPs that were likely to alter ARNT function. Based on HapMap data and our own genotyping, the selected tagSNPs should have adequately covered all common genetic variants. No SNP showed an allelic association with type 2 diabetes, either European American or African American individuals. Although we found associations of SNPs rs2134688 (intron 4) and rs4379678 (3' flanking region) under a recessive model, these findings could not be confirmed in a larger population and we believe the original observations, which were also out of Hardy Weinberg Equilibrium, were likely spurious and due to small sample size. We anticipated that variation in *ARNT *would alter insulin secretion, and we indeed found evidence for an association of SNPs rs188970 (a perfect proxy for synonymous SNP rs2228099) and rs11204735, with altered AIR_g _among members of high risk families. On the other hand, these findings were not replicated in a separate and differently ascertained population from Arkansas. The lack of replication may reflect differences in the two populations – one cohort of related individuals from high risk families, the other a more heterogeneous and unrelated population from Arkansas with more obese subjects and a very different environment. Alternatively, the observation in high risk families may have been spurious, as we tested 3 traits and 4 SNPs. However, the suspected biology of *ARNT *would suggest that environmental interactions may differ across populations. The same arguments apply for the interaction of SNP rs2134688 with gender to determine DI. Additional studies are needed to determine the role of these variants in insulin secretion.

Replication of genetic associations, both with dichotomous traits such as type 2 diabetes and quantitative traits such as AIR_g _has been difficult with sample sizes that are practical for individual laboratories. We have sought alternative methods based on comparison of *ARNT *expression among siblings discordant for type 2 diabetes, and using allelic expression imbalance for transcribed SNPs to find evidence for regulatory variants [13]. In the current study, we examined two transcribed SNPs. In both cases, a few heterozygous individuals showed allelic imbalance outside of the range observed for genomic DNA (Figure 2), suggesting a possible regulatory mutation. Whether these results in transformed lymphocytes reflect expression in pancreatic β-cells is unknown, and the two SNPs appear to give different results. Nonetheless, SNP rs2228099 is in strong linkage disequilibrium with the SNP rs188970 that was associated with altered insulin secretion.

The strengths of this study are the use of multiple approaches to examine the *ARNT *gene, including association studies in two populations, quantitative trait studies for insulin secretion, and gene expression studies in transformed lymphocytes as a surrogate tissue for pancreatic β-cells. Nonetheless, this study had several limitations. First, the association study was of modest size, and would have been unable to detect a role for variants with small effect. Recent genome wide association scans in type 2 diabetes [20-23] have suggested that most if not all loci had odds ratios (ORs) below 1.5 and generally near 1.2 or less. Whereas we had 70% power to detect an association for common SNPs with an OR in the range of 1.5–1.7, or the approximate effect of the *TCF7L2 *gene [24], we had very limited power to detect effects with an OR of 1.2 or less. Although data for most published genome wide association scans are not yet publicly available, the Diabetes Genome Initiative scan [23] data are publicly available, and show 3 SNPs in the ARNT gene that can be estimated to capture 50% of the genetic variation. As with our study, no SNP showed any trend to association in over 1400 cases and 1400 controls. Based on allelic association and p < 0.05, the two SNPs at 35% minor allele frequency had 87% power to exclude an odds ratio of 1.2 or greater. That effect size would be comparable to the role of known diabetes genes including the potassium channel gene *KCNJ11 *E23K variant, or peroxisome proliferator activating receptor γ (*PPARG*) P12A variant, or more recently described variants that have required very large populations to confirm [20-23,25]. Hence, our failure to detect an association with type 2 diabetes in our more modest sample size (albeit with two separately examined study populations) probably did not result from SNPs of small effect size.

Small effects on insulin secretion similarly would not have been detectable in populations of a size that could be examined with detailed phenotyping, as was done in our study. Thus, additional *ARNT *variants might have a small effect on insulin secretion that we did not detect, or might have an effect on aspects of insulin secretion that we did not measure. Finally, we did not exhaustively screen the *ARNT *gene for variants, but instead used a combination of screening conserved regions and indirect screening based on linkage disequilibrium relationships to test the common variants. Although unlikely, a causative SNP outside of the region screened and not in linkage disequilibrium with our tagSNPs might have gone undetected.

## Conclusion

Gunton et al. [3] observed a 90% decrease in *ARNT *expression in pancreatic islets from type 2 diabetic individuals when compared with nondiabetic islets. In contrast, we found no difference in *ARNT *message in transformed lymphocytes from siblings with type diabetes and nondiabetic siblings. Our results may reflect the lack of correspondence of gene expression in transformed lymphocytes and β-cells, or Gunton et al may have found expression changes that were secondary to hyperglycemia. Our data provide some evidence that *ARNT *variants may alter insulin secretion and that *ARNT *gene variants may alter transcript levels, but our findings are not compatible with the dramatic differences in transcript levels observed by Gunton et al. We suggest that such striking reductions in *ARNT *transcript levels in the diabetic pancreas are unlikely to be caused by genetic variants and thus not the direct cause of the insulin secretory defect that occurs early in type 2 diabetes and progresses throughout the disease.

## Competing interests

The author(s) declare that they have no competing interests.

## Authors' contributions

SKD directed the project, was primarily responsible for data interpretation, SNP selection, sequence analysis, and gene expression in sibling pairs, and wrote the first draft of the manuscript. NKS performed genotype analysis. WSC assisted with genotyping and prepared all samples for analysis. HW performed allelic imbalance studies. SCE conceived the project design with SKD, performed all analyses of insulin secretion and sensitivity, oversaw and reviewed all other analyses, oversaw all human subject studies, performed all MinMod analyses, and prepared the final draft of the manuscript. All authors read and approved the final manuscript.

## Pre-publication history

The pre-publication history for this paper can be accessed here:


